# Missing data imputation, prediction, and feature selection in diagnosis of vaginal prolapse

**DOI:** 10.1186/s12874-023-02079-0

**Published:** 2023-11-06

**Authors:** Mingxuan FAN, Xiaoling Peng, Xiaoyu Niu, Tao Cui, Qiaolin He

**Affiliations:** 1 Guangdong Provincial Key Laboratory of Interdisciplinary Research and Application for Data Science, BNU-HKBU United International College, Zhuhai, 519087 China; 2 Department of Gynecology and Obstetrics, West China Second University Hospital, Sichuan University, Chengdu, 610064 China; 3 Key Laboratory of Birth Defects and Related Diseases of Women and Children (Sichuan University), Ministry of Education, Chengdu, 610064, China; 4 School of Mathematics, Sichuan University, Chengdu, 610064, China

**Keywords:** Missing data imputation, Generative adversarial imputation, Feature selection, Classification, Diagnosis of vaginal prolapse

## Abstract

**Background:**

Data loss often occurs in the collection of clinical data. Directly discarding the incomplete sample may lead to low accuracy of medical diagnosis. A suitable data imputation method can help researchers make better use of valuable medical data.

**Methods:**

In this paper, five popular imputation methods including mean imputation, expectation-maximization (EM) imputation, K-nearest neighbors (KNN) imputation, denoising autoencoders (DAE) and generative adversarial imputation nets (GAIN) are employed on an incomplete clinical data with 28,274 cases for vaginal prolapse prediction. A comprehensive comparison study for the performance of these methods has been conducted through certain classification criteria. It is shown that the prediction accuracy can be greatly improved by using the imputed data, especially by GAIN. To find out the important risk factors to this disease among a large number of candidate features, three variable selection methods: the least absolute shrinkage and selection operator (LASSO), the smoothly clipped absolute deviation (SCAD) and the broken adaptive ridge (BAR) are implemented in logistic regression for feature selection on the imputed datasets. In pursuit of our primary objective, which is accurate diagnosis, we employed diagnostic accuracy (classification accuracy) as a pivotal metric to assess both imputation and feature selection techniques. This assessment encompassed seven classifiers (logistic regression (LR) classifier, random forest (RF) classifier, support machine classifier (SVC), extreme gradient boosting (XGBoost) , LASSO classifier, SCAD classifier and Elastic Net classifier)enhancing the comprehensiveness of our evaluation.

**Results:**

The proposed framework imputation-variable selection-prediction is quite suitable to the collected vaginal prolapse datasets. It is observed that the original dataset is well imputed by GAIN first, and then 9 most significant features were selected using BAR from the original 67 features in GAIN imputed dataset, with only negligible loss in model prediction. BAR is superior to the other two variable selection methods in our tests.

**Concludes:**

Overall, combining the imputation, classification and variable selection, we achieve good interpretability while maintaining high accuracy in computer-aided medical diagnosis.

## Introduction

Pelvic organ prolapse (POP) is a major health issue for women, which is defined as the descent of one or more pelvic organs, such as the anterior and posterior vaginal wall, uterus (cervix), or apex of the vagina (vaginal vault or cuff scar after hysterectomy). POP significantly impacts the quality of life, causing discomfort, pain, and embarrassment. The cause of POP is multifaceted, and the known risk factors include aging, menopause, parity, vaginal delivery, weight lifting, obesity, and chronic cough [1]. The prevalence of symptomatic POP in China is 9.6%, and the incidence of POP in women aged 70 years or older is reported to be eight times higher than that in women aged 20-29 years old [2]. The lifetime risk of surgery for POP is 11.1% [3]. Despite the high incidence of POP, its pathophysiological mechanism has not been elucidated. Pelvic floor disorders, including pelvic organ prolapse (POP), urinary incontinence and fecal incontinence, are common ailments in middle-aged and elderly women. In POP, prolapse of the anterior vaginal wall is the most frequent form. Despite the high incidence, multiple factors such as inadequate awareness and shyness lead to a low clinic rate, making data collection harder and incomplete. Therefore, it’s important to figure out how to utilize the clinical data and minimize the impact of data loss. The issue of missing data in the diagnosis of vaginal prolapse is a concern due to privacy issues, as some patients may map out of sharing certain information. The missing data can lead to potential misdiagnosis, which can have significant clinical implications. Deletion of samples with missing values, also known as complete case analysis or listwise deletion, is a commonly used approach for handling missing data. However, this approach can introduce substantial bias if the missing data are not missing completely at random, especially when the deleted values are related to the outcome variables [4, 5]. In these scenarios, imputation methods are often preferred over deletion. Two broad categories of imputation methods are typically used: statistical and machine learning methods [6].

Statistical methods deal with missing values by filling the missing part with its statistical estimate calculated from the available part [7], which include expectation maximization (EM) [8], gaussian mixture model (GMM) [9], Hot deck imputation [10], linear discriminant analysis (LDA) [11], Markov Chain Monte Carlo (MCMC) [12], Mean/Mode imputation [6], Multiple Imputation by Chained Equations (MICE) [13], Naive Bayes [14, 15], Principal Component Analysis (PCA) [16] and Singular Value Decomposition (SVD) [17]. In this paper we only research on mean/mode imputation and expectation maximum imputation because they’re commonly used and typical. Because of its simplicity and ease of computation, the mean imputation, which replaces missing values with the mean of the available observations, is the most commonly used imputation method. As the the sample mean is sensitive to extreme values, median or mode can be used as alternate when the data distribution is not normal. However, single imputation does not take into account the research objectives and individual differences, which may largely impact the estimation accuracy [18]. So it is important to carefully develop the appropriate imputation method based on the characteristics of the data and the research question being addressed. More sophisticated methods like multiple imputation that combine multiple estimates from a suitable imputation model can reduce the bias and narrow the uncertainty. However, doing multiple imputation well can be a tough task, since choosing and applying a suitable imputation model requires knowing well your data set [19]. EM imputation iteratively finds the maximum likelihood estimates via E-step and M-step . It is flexible that can be applied to a wide range of cases and relatively simple to be implemented. Many studies show that EM imputation is effective in various statistical models [20, 21], although it is sensitive to initial values. Additionally, EM algorithm can converge to a local maximum, which means that it may not always find the global maximum likelihood estimate [22].

Compared with statistical methods, machine learning techniques excel at exploring complex relationships between large data sets.

On the other hand, the realm of machine learning offers an array of powerful methods for data imputation. Artificial Neural Networks are capable of learning complex patterns in data and predicting missing values [23]. Association rule mining identifies associations between variables that can aid in imputation. Clustering algorithms, such as Fuzzy C-Means and K-Means, group data points to facilitate imputation [24, 25]. Self-Organizing Maps (SOM) provide dimensionality reduction for accurate imputation. Decision tree-based methods like Classification and Regression Trees (CART) and C4.5 offer intuitive ways to predict missing values based on available information [26]. K-Nearest Neighbor (KNN) imputation leverages proximity to similar data points for estimation [27]. Kernel-based imputation and Support Vector Machine/Regression (SVM/SVR) techniques further extend the repertoire of imputation methods within the machine learning framework [28]. In recent years, machine learning methods such as denoising autoencoders (DAE) [29], and generative adversarial imputation nets (GAIN) [30] have become increasingly popular in dealing with incomplete data. In our paper, we will focus on KNN, DAE and GAIN, because KNN is commonly used and GAIN, DAE are relatively new methods, according to our experiments, they are better suited to the real dataset applied in this paper.

KNN imputation replaces the missing value with the mean or mode of its nearest neighbours [31]. Although KNN performs well on some public data sets [27], it is very complex to compute, especially in high-dimensional data, which is a drawback that makes it hard to be applied in real situation.

To reduce the complexity, a more practical approach applying Self-Organizing Map (SOM) was proposed [32]. DAE was first proposed as a new training principle for unsupervised learning [33]. Inspired by this, *Gondara et al.* apply DAE in missing data imputation , this method is valid for different types of missing data [29]. Another deep learning network be more appropriate for complex, high-dimensional distributions. *Ozair et al.* demonstrated the power of Generative adversarial networks in imputation with comprehensive simulations [34]. Unlike likelihood-based methods, GANs are considered as implicit probabilistic models [35]. Later on, an imputing method GAIN was proposed by adapting the GAN framework [30]. Yoon et al. showed that GAIN outperformed the other five imputation methods including multivariate imputation by chained equations (MICE) [36], MissForest [37], Matrix [38], DAE and EM imputation [30].

In data-driven medical diagnosis, it is also crucial to automatically pick out the major risk factors for certain disease among a large number of candidate indicators [39]. To address this issue, many variable selection techniques have been utilized to select the most relevant features, enhance model interpretability and avoid overfitting [40]. In principle, exhaustive searching of all possible combinations of variables is an ideal way for selecting the best subset. But this method will be computational infeasible when the number of variables *d* is large. Since LASSO [41], variable selection via regularized regression has been one of the hot topics in many real applications including medical data analysis. Such regularized models can identify most relevant variables and estimate regression coefficients simultaneously. In past years, various penalty functions have been employed in regularization for variable selection. As the first proposed regularization regression, LASSO utilizes $L_1$ penalty which produces biased estimates for large coefficients. This has motivated Fan and Li [42] to consider a superior penalty, the smoothly clipped absolute deviation (SCAD) penalty. They proved that the SCAD has three properties for the penalty function: sparsity, unbiasedness and continuity. Also, the Elastic Net [43] which was proposed as the combination of L1 and L2 penalty, is very robust and less biased compared to LASSO. Theoretically, $L_0$ regularized regressions which directly penalize the number of nonzero parameters, should be the most essential sparsity measure. However, solving an $L_0$ regularized optimization is quite challenging because of its lack of convexity. In order to approximate the $L_0$ regularization in generalized linear models (GLM), broken adaptive ridge (BAR) was proposed using an iterative reweighed $L_2$-penalization procedure [44]. The GLM-BAR estimator possesses the advantages of both $L_0$ and $L_2$ penalizations and comparing to $L_1$ penalization, it tends to generate more concise model [44].

In this article, we develop a general framework which connects data imputation, prediction and feature selection. Taking the vaginal prolapse data set as an example, we first compare five imputation methods (mean imputation, EM imputation, GAIN, KNN imputation, and DAE) for their performance of handling missing data. Then LASSO, SCAD, BAR are applied on imputed data sets for feature selection and classification. These methods, in themselves, do not possess classification function. Their classification function only emerges when applied to a specific generalized model, such as logistic regression. So we work on some combinations in this work. Our contributions in this work are:

- Through real medical data, we successfully demonstrated that missing value imputation can greatly improve the prediction accuracy in clinical diagnosis.
- Imputation and feature selection are combined and adapted to the specific area of vaginal prolapse prediction for the first time. We integrate them synergistically to leverage the strengths of both methods within the unique characteristics of our dataset.
- The proposed framework imputation-variable selection-prediction is applicable to most medical diagnosis based on incomplete datasets.

The paper is organized as follows. In “Methodology” section, specific details are provided about the five imputation methods and the three feature selection methods. Then we show experimental results of imputation, feature selection and classification in “Results” section. Conclusions and discussions are presented in “Conclusions and discussions” section.

## Methodology

### Mean imputation

In this approach, missing components of every attribute are filled in by the average of all observed components in corresponding attribute [4].

### EM algorithm

It assumes that the input vectors are generated from some probability distribution function (pdf) $p(x|\theta )$, where $\theta$ is the parameter which determines this probability distribution. Given $x_1,..., x_N$, the observed *d*-dimensional predictors, $l(\theta )=\sum _{i=1}^N \log p(x_i|\theta )$ is the log-likelihood function of $\theta$. If there exists hidden data *z* with distribution *Q*(*z*), then $p(x_i|\theta )=\sum _{z}p(x_i,z|\theta )$ and the log-likelihood function becomes:

1 $$\begin{aligned} l(\theta ){} & {} =\sum \limits _{i=1}^{N}\log \sum \limits _{z}p(x_i,z|\theta ) \nonumber \\{} & {} =\sum \limits _{i=1}^{N}\log \sum \limits _{z}Q(z)\frac{p(x_i,z|\theta )}{Q(z)}. \end{aligned}$$

According to Jensen’s Inequality (2), we have

2 $$\begin{aligned} l(\theta ){} & {} = \sum \limits _{i=1}^{N}\log \sum \limits _{z}Q(z)\frac{p(x_i,z|\theta )}{Q(z)} \nonumber \\{} & {} \ge \sum \limits 7_{i=1}^{N}\sum \limits _{z}\log Q(z)\frac{p(x_i,z|\theta )}{Q(z)}. \end{aligned}$$

The lower bound of $l(\theta )$ reaches its maximum when the equality holds, that is,

3 $$\begin{aligned} Q(z){} & {} = p(z|x_i,\theta ). \end{aligned}$$

With an initialized distribution parameter $\theta$, the following two steps (E and M) are repeated until convergence.

- E step: compute the conditional probability expectation of the joint distribution.
  4 $$\begin{aligned} Q(z){} & {} = p(z|x_i,\theta ), \end{aligned}$$
  5 $$\begin{aligned} E\left(\frac{p(x_i,z|\theta )}{Q(z)}\right){} & {} =\sum \limits _{i=1}^{N}\sum \limits _{z}\log Q(z)\frac{p(x_i,z|\theta )}{Q(z)}. \end{aligned}$$
- M step: maximize the expectation.
  6 $$\begin{aligned} \theta{} & {} = \arg \max E\left( \frac{p(x_i,z|\theta )}{Q(z)}\right) . \end{aligned}$$

### KNN

KNN imputation is a non-parametric method that does not make assumptions about the underlying distribution of the data. Based on KNN, the missing values are imputed by the mean or mode of their nearest neighbors which are defined as the *K* closest data points to the missing value according to some distance metric [31]. Choosing appropriate *K* - the number of neighbors and distance function are two important issues in KNN imputation. Troyanskaya et al. suggested to use non-missing part to calculate optimal *K* [45]. But until now, there is no theoretical optimal *K*. As to the distance between samples, Minkowski distance is used in general. Assume that observed samples have *d* attributes, for example, the i-th sample is represented as $x_i=(x_{i1},x_{i2},...,x_{id})$. Then the Minkowski distance between two samples $x_i$ and $x_j$ is defined in Eq. (7).

7 $$\begin{aligned} D(x_i,x_j){} & {} =(|x_{i1}-x_{j1}|^q+|x_{i2}-x_{j2}|^q+...\nonumber \\{} & {} +|x_{id}-x_{jd}|^q)^{\frac{1}{q}}, \end{aligned}$$

where *q* is the Minkowski coefficient. In this paper, we use Euclidean distance, which is a special case when the parameter $q=2$.

### DAE

DAE, a deep neural network, is composed of an encoder and a decoder, where both the encoder and decoder are three-layer neural networks. The decoder is first given the input *d*-dimensional with missing data, and the mean value of the corresponding variable is used as a placeholder for the missing position. The units in each layer of the encoder is $d+\theta ,d+2\theta ,d+3\theta$, and the units in each layer of the decoder is $d+3\theta , d+2\theta , d+\theta$, and the final output is a d-dimensional complete data. $\theta$ represents positive integer, which in this article we set it to 7. DAE is implemented based on Python 3.9.0.

### GAIN

**Fig. 1:**
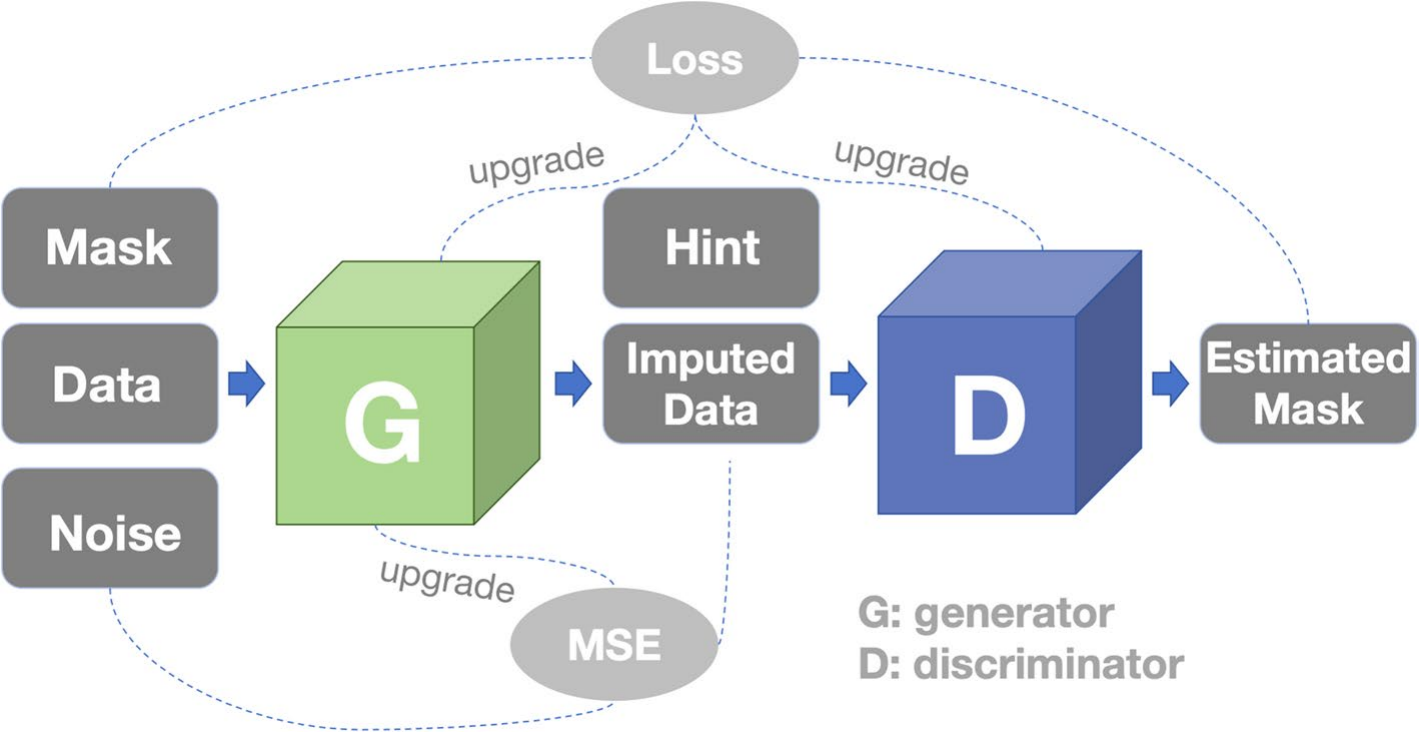
Architecture of GAIN

Proposed by Yoon, J et al., GAIN imputes missing data using well-known GAN framework [30]. The generator (G) observes some components of a real data vector, imputes the missing components conditioned on what is actually observed, and outputs a completed vector. The discriminator (D) then takes a completed vector and attempts to determine which components were actually observed and which were imputed. To ensure that D forces G to learn the desired distribution, D is provided with some additional information in the form of a hint vector. The hint reveals to D partial information about the missing part of the original samples, which is used by D to focus its attention on the imputation quality of particular components. This hint ensures that G is in fact generated according to the true data distribution. The architecture of GAIN algorithm is shown in Fig. 1. We define some random vectors: data vector $X=(X_{1} ,..., X_{d})$, mask vector $M=(M_{1} ,..., M_{d})$ and noise vector $Z=(Z_{1} ,..., Z_{d})$, where *M* takes values in $\{0,1\}^d$.

$$\begin{aligned} M_i=\left\{ \begin{array}{ll} 1&{} \text {if}\ X_i \ \text {is observed},\\ 0&{} \text {otherwise}. \end{array}\right. \end{aligned}$$

#### Generator

The generator (G) is a fully connected neural network. Its output $\bar{X}$ is

8 $$\begin{aligned} \bar{X}=G(X, M, (\varvec{1}-M)\odot Z), \end{aligned}$$

where $\odot$ represents element-wise multiplication and $\varvec{1}$ denotes the unit vector. Then the overall output $\hat{X}$ can be generated as:

9 $$\begin{aligned} \hat{X}=M\odot X+(\varvec{1}-M)\odot \bar{X}. \end{aligned}$$

#### Discriminator

The discriminator (D) is also a fully connected neural network mapping imputed data into $[0,1]^d$, where the *i*-th component represents the possibility that the *i*-th component of $\hat{X}$ is observed.

#### Hint matrix

To ensure that enough information is provided to D, a hint mechanism is necessary [30].Consider a random variable $B=(B_1 ,..., B_d)$,

$$\begin{aligned} B_j= \left\{ \begin{array}{ll} 0&{} {j=k},\\ 1&{} {j\ne k}. \end{array}\right. \end{aligned}$$

where $k \in \{1,2...,d\}$ is randomly selected. The hint vector *H* is then defined based on *B*:

10 $$\begin{aligned} H{} & {} = B\odot M+0.5(\varvec{1}-B). \end{aligned}$$

#### Loss function

To obtain the object of adversarial training, a quantity is defined in Eq. (11)

11 $$\begin{aligned} V(D,G){} & {} =E_{\hat{x},M,H}[(\varvec{1}-M)^T \log (\varvec{1}-D(\hat{X},H))\nonumber \\{} & {} +M^T \log D(\hat{X},H)], \end{aligned}$$

then GAIN is simplified as a minimax problem

12 $$\begin{aligned} \min \limits _{G} \max \limits _{D}V(D,G). \end{aligned}$$

Based on this problem, the loss function is given as

13 $$\begin{aligned} \mathcal {L}(a,b){} & {} = \sum \limits _{i=1}^d[a_i\log (b_i)+(1-a_i)\log (1-b_i)], \end{aligned}$$

and the loss function of D is then defined as

14 $$\begin{aligned} \mathcal {L}_D(m,\hat{m},b)&=\sum \limits _{i:b_i=0}[(1-m_i)\log (1-\hat{m_i})\nonumber \\&+m_i \log (\hat{x_i})]. \end{aligned}$$

It is obvious to see that D is updated by

15 $$\begin{aligned} \min \limits _{D}-\sum \limits _{j=1}{k_D}\mathcal {L}_D(m(j),\hat{m}(j),b(j)), \end{aligned}$$

where $k_D$ is the mini batch size. Similarly, the loss function of G is defined as follows

16 $$\begin{aligned} \mathcal {L}_G(m,\hat{m},b){} & {} = -\sum \limits _{i:b_i=0}(1-m_i)\log (\hat{m_i}). \end{aligned}$$

By reducing the loss in Eq. (16), the generator can successfully obfuscate the discriminator. Since the part generated by the generator should be as close to the real data as possible, another loss function Eq. (17) is defined as below to guarantee the similarity:

17 $$\begin{aligned} \mathcal {L}_M(x_i,x_i')=\sum \limits _{i=1}^d m_iL_M\left(x_i,x_i'\right), \end{aligned}$$

where

$$\begin{aligned} L_M(x_i,x_i')= \left\{ \begin{array}{ll} \left(x_i'-x_i\right)^2,&{} \text {if}\ x_i\ \text {is continuous},\\ -x_i\log {x_i'}.&{} \text {if}\ x_i\ \text {is binary}. \end{array}\right. \end{aligned}$$

The G is updated by

18 $$\begin{aligned}{} & {} \min \limits _{G}\sum \nolimits _{j=1}^{k_G}\mathcal {L}_G(m(j),\hat{m}(j),b(j))\nonumber \\{} & {} \quad \quad \quad \quad +\alpha \mathcal {L}_M(x(j),\hat{x}(j)), \end{aligned}$$

where $k_G$ is the mini batch size, $\alpha$ is a hyper parameter. In this study, GAIN is implemented based on Python 3.9.0.

### Variable selection in generalized linear models

For generalized linear models such as ordinary linear regression, logistic regression and Poisson regression, let $y_1,y_2,...,y_N$ be *N* observations of response variables and $\varvec{x}_1$, ..., $\varvec{x}_N$ be observed *d*-dimensional predictors corresponding to $y_1,y_2,...,y_N$. Each $y_i$ has the exponential distribution:

19 $$\begin{aligned} f_Y(y_i|\theta _i,\phi ){} & {} = \exp \left\{ \frac{y_i \theta _i-a(\theta _i)}{b(\phi )}-c(y_i,\phi )\right\} , \end{aligned}$$

where $\phi \ (0<\phi <\infty )$, $\theta _i$ are parameters, $a(\cdot ), b(\cdot )$ and $c(\cdot )$ are known functions. Through a specified link function $h(\cdot )$, $\theta _i$ is connected with $\mathbf {x_i}$ as $h(\mu _i)=\mathbf {x_i}^T\beta$, where $\mu _i$ is the expectation of $y_i$ that can be obtained by $\mu _i=a'(\theta _i)$, and $\beta =\{\beta _1,...,\beta _p\}$ are regression coefficients of the GLM. Then the log-likelihood function is

20 $$\begin{aligned} l(\beta ){} & {} = \sum \limits _{i=1}^n \log L(\beta ;\mathbf {x_i},y_i) \nonumber \\{} & {} = \sum \limits _{i=1}^n \log f_Y(y_i;\theta _i,\phi ). \end{aligned}$$

Adding a penalty to the log-likelihood function will provide both variable selection and regression coefficient estimation for GLMs by simultaneously identifying a subset of significant variables. The $\beta$ estimator is computed by minimizing an objective function combining the goodness of fit and sparsity.

21 $$\begin{aligned} \hat{\beta }=\arg \min _{\beta }\{-l(\beta )+P_{\lambda }(\beta )\}. \end{aligned}$$

Three typical penalty functions commonly used for variable selection are described briefly below.

#### LASSO

The well known variable selection approach shrinks some small coefficients using $L_1$ penalty  

22 $$\begin{aligned} P_{\lambda }(\beta )=|\beta |, \end{aligned}$$

where $\lambda$ is a non-negative tuning parameter for model sparsity.

#### SCAD

 

23 $$\begin{aligned} P_{\lambda }(\beta ){} & {} = \lambda \left\{ I(\beta <\lambda )+\frac{(a\lambda -t)_+}{(a-1)\lambda }I(\beta >\lambda )\right\} . \end{aligned}$$

LASSO and SCAD are both implemented by R package *glmnet*.

#### BAR

The BAR method is proposed to approximate the $L_0$ penalty. Initialized with the solution of ridge regression, the GLM-BAR estimator of $\beta$ is updated by reweighed $L_2$-penalized regression ($k\ge 1$)

24 $$\begin{aligned} \hat{\beta }^{(k)}{} & {} = \arg \min _{\beta }\left\{ -l(\beta )+\frac{\lambda }{2}\sum \limits _{j=1}^p\frac{\beta _j^2}{\left(\hat{\beta _j}^{(k-1)}\right)^2}\right\} . \end{aligned}$$

In this study, BAR is implemented using R BrokenAdaptiveRidge package .

## Results

### Data description

The vaginal prolapse data set were collected through the specialized database of Pelvic Floor Dysfunction, from a National Key R &D Program of China, at West China Second University Hospital. The data set analyzed in this study includes demographic, therapeutic and recurrence-survival information of 28,274 women with and without POP from January 1, 2015 to December 31, 2021.

**Fig. 2:**
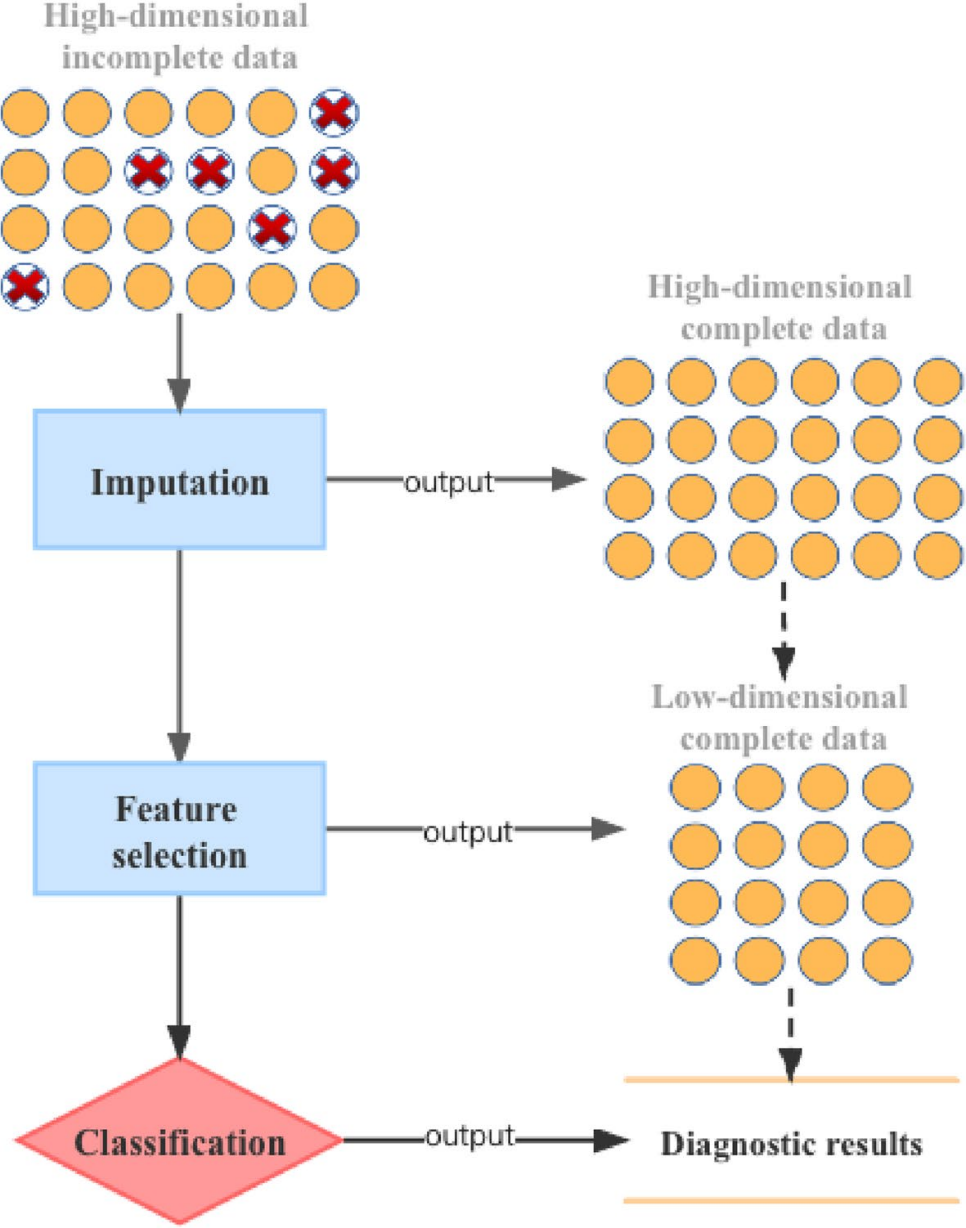
Process of missing data imputation, prediction, and feature selection in data-driven medical diagnosis

After primary data cleaning, 12694 valid individuals with 67 prognostic factors are remained in our study. In this data matrix, the total missing rate reaches $7.71\%$. In all valid cases, 9840 of them contain at least one missing attributes, representing $77.52\%$ of all 12694 cases. Table 1 shows the basic information of the five features with the highest missing rate, such as mean, range and the proportion of missing values. If all these incomplete items are deleted before modeling, we will lose a lot of useful information, resulting in inaccurate prediction and diagnosis of the disease. Therefore, choosing appropriate data imputation method to fully utilize existing data information is crucial for solving such data-driven medical diagnosis problems. On the other side, how to select the important factors that are truly related to the disease from 67 potential variables is also a matter of great concern to doctors. In this work, a general framework which connects data imputation, prediction and feature selection is proposed and presented in Fig. 2.

**Table 1:** The five variables with highest missing rate

Factor	Range	Mean	Missing rate (%)
Prerest pelvicfloor mean	0-112.6	6.77	30.7
Fast muscle rise	0.14-2	0.43	30.6
Incontinence(mom or sis)	1-2	1.01	23.2
Stress incontinence	1-2	1.07	23.1
Vaginal laxity	1-4	1.63	22.8

### Evaluation criteria

To assess the efficacy, classification models LR, RF, SVC, XGBoost, LASSO based classifier (LASSO-LR), SCAD based classifier (SCAD-LR) and Elastic Net based classifier (Elastic Net-LR) are implemented on each imputed data [46]. The accuracy, $F_1$ score and AUC are used as the performance metrics for evaluating the proposed imputation methods. For each classifier, we repeated the experiment 100 times and show the averaged results and standard deviation.

- True Positives (TP): Instances correctly classified as positive.
- True Negatives (TN): Instances correctly classified as negative.
- False Positives (FP): Instances incorrectly classified as positive.
- False Negatives (FN): Instances incorrectly classified as negative.
- **Accuracy**: ratio of the number of correct classifications to the total number.
  $$\begin{aligned} \text {Accuracy} = \frac{\text {True Positives} + \text {True Negatives}}{\text {True Positives} + \text {True Negatives} + \text {False Positives} + \text {False Negatives}} \end{aligned}$$
- $\mathbf {F_1}$ **score**: the summed average of precision and recall, with maximum of 1 and minimum of 0.
  $$\begin{aligned} F_1 = \frac{2\times \textrm{precision}\times \textrm{recall}}{\text {precision+recall}} \end{aligned}$$
  where
  $$\begin{aligned} precision = \frac{TP}{TP + FP} \end{aligned}$$
  $$\begin{aligned} recall = \frac{TP}{TP + FN} \end{aligned}$$
- **AUC**: the area under the receiver operating characteristic (ROC) curve.

### Prediction without imputation and feature selection

In order to better emphasize the effect of imputation and feature selection, we will utilize the dataset without imputation and without feature selection, that is, only case deletion is used on the original dataset. We will then showcase performance metrics, including accuracy, F1 score, and AUC (Area Under the Curve), under these conditions. Classification results are displayed in Table 2. It appears that the performance of LD is not very satisfactory, as its accuracy, F1 score, and AUC all hover around 70%. This suggests that LD may not be effectively capturing the underlying patterns in the data or that there may be room for improvement in its performance.

**Table 2:** Classification results of LD dataset

Classifier	Accuracy	F1	AUC
**LR**	0.7002(0.0029)	0.7252(0.0030)	0.6975(0.0031)
**RF**	0.6826(0.0261)	0.7038(0.0276)	0.6810(0.0265)
**SVC**	0.5989(0.2220e-16)	0.5831(0.0)	0.6459(0.5310e-5)
**XGBoost**	0.6551(0.0180)	0.6762(0.0201)	0.7127(0.0191)
**LASSO-LR**	0.6935(0.0177)	0.7187(0.0182)	0.6909(0.0180)
**SCAD-LR**	0.6861(0.0179)	0.7131(0.0191)	0.6833(0.0177)
**Elastic Net-LR**	0.6943(0.0181)	0.7196(0.0184)	0.6917(0.0184)

### Imputation

To show whether the feature selection strategies were useful, we display the accuracy, F1, and AUC by just using LR, RF, SVC, XGBoost, LASSO-LR, SCAD-LR, and Elastic Net-LR on the dataset without feature selection and with data imputation in this section. Tables 3, 4, 5, 6, 7, 8 and 9 displays the classification results of the classifiers mentioned in “Evaluation criteria” on the imputed datasets, respectively. It is observed that the accuracy of all imputation methods is higher than that of LD method. It is evident that RF classifier shows better performance than other three classifiers when imputed datasets are used. Furthermore, GAIN achieves superior performance compared to the other methods across all four classifiers.

**Table 3:** Classification results of imputed datasets under LR classifier

Imputation Method	Accuracy	F1	AUC
**Mean**	0.7164(0.0012)	0.8110(0.0008)	0.6158(0.0025)
**EM**	0.7148(0.0011)	0.8098(0.0008)	0.6140(0.0025)
**KNN**	0.7174(0.0013)	0.8113(0.0009)	0.6179(0.0024)
**DAE**	0.7170(0.0015)	0.8030(0.0011)	0.6434(0.0033)
**GAIN**	**0.8010(0.0009)**	**0.8555(0.0006)**	**0.7641(0.0015)**

**Table 4:** Classification results of imputed datasets under SVC

Imputation Method	Accuracy	F1	AUC
**Mean**	0.7113(<e-33)	0.8177(0.1232e-31)	0.7595(<e-33)
**EM**	0.6955(<e-33)	0.8204(<e-33)	0.7750(0.1232e-31)
**KNN**	0.7715(0.1232e-31)	0.8445(0.4930e-31)	0.8201(<e-33)
**DAE**	0.7101(<e-33)	0.8150(<e-33)	0.7641(1.1093e-31)
**GAIN**	**0.7948(0.4930e-31)**	**0.8511(0.1232e-31)**	**0.8666(<e-33)**

**Table 5:** Classification results of imputed datasets under XGBoost classifier

Imputation Method	Accuracy	F1	AUC
**Mean**	0.7874(0.0075)	0.8443(0.0061)	0.8563(0.0069)
**EM**	0.7846(0.0073)	0.8440(0.0058)	0.8490(0.0068)
**KNN**	0.7862(0.0074)	0.8441(0.0059)	0.8519(0.0065)
**DAE**	0.7007(0.0082)	0.7868(0.0067)	0.7562(0.0084)
**GAIN**	**0.7894(0.0072)**	**0.8456(0.0057)**	**0.8569(0.0067)**

**Table 6:** Classification results of imputed datasets under RF classifier

Imputation Method	Accuracy	F1	AUC
**Mean**	0.7937(0.0072)	0.8504(0.0058)	0.7566(0.0084)
**EM**	0.7892(0.0082)	0.8499(0.0065)	0.7412(0.0092)
**KNN**	0.7916(0.0073)	0.8495(0.0055)	0.7516(0.0095)
**DAE**	0.7030 (0.0069)	0.7899(0.0058)	0.6371(0.0073)
**GAIN**	**0.8064(0.0101)**	**0.8510(0.0082)**	**0.7614(0.0118)**

**Table 7:** Classification results of imputed datasets under LASSO-LR classifier

Imputation Method	Accuracy	F1	AUC
**Mean**	0.7098(0.0077)	0.8082(0.0059)	0.6026(0.0070)
**EM**	0.7096(0.0075)	0.8082(0.0059)	0.6020(0.0067)
**KNN**	0.7120(0.0074)	0.8094(0.0057)	0.6062(0.0071)
**DAE**	0.7078(0.0080)	0.7985(0.0064)	0.6274(0.0089)
**GAIN**	**0.7957(0.0069)**	**0.8521(0.0055)**	**0.7577(0.0081)**

**Table 8:** Classification results of imputed datasets under SCAD-LR classifier

Imputation Method	Accuracy	F1	AUC
**Mean**	0.7104(0.0080)	0.8087(0.0060)	0.6031(0.0076)
**EM**	0.7099(0.0073)	0.8084(0.0056)	0.6027(0.0068)
**KNN**	0.7127(0.0074)	0.8098(0.0057)	0.6075(0.0073)
**DAE**	0.7007(0.0085)	0.7987(0.0066)	0.6030(0.0082)
**GAIN**	**0.7923(0.0069)**	**0.8504(0.0054)**	**0.7505(0.0083)**

**Table 9:** Classification results of imputed datasets under elastic Net-LR classifier

Imputation Method	Accuracy	F1	AUC
**Mean**	0.7111(0.0082)	0.8094(0.0063)	0.6032(0.0075)
**EM**	0.7103(0.0082)	0.8089(0.0064)	0.6024(0.0074)
**KNN**	0.7122(0.0080)	0.8098(0.0062)	0.6058(0.0075)
**DAE**	0.7078(0.0081)	0.7980(0.0066)	0.6287(0.0086)
**GAIN**	**0.7976(0.0073)**	**0.8536(0.0060)**	**0.7590(0.0085)**

### Feature selection

As stated in “Methodology” section, we implement three feature selection methods on individual imputed dataset via logistic regression to select most relevant factors of the vaginal prolapse. These methods include LASSO, SCAD and BAR. It is worth mentioning that the original LASSO tends to selected more variables. However, the algorithm in the R package we are using now has a very practical improvement in providing strong rules that can screen out a large number of predictors, yet producing sparse solutions [47].

**Table 10:** Classification results of datasets after feature selection under LR classifier

Imputation methods	FSM(No. of selected features)	Accuracy	F1	AUC
**Mean**	LASSO(20)	0.7132(0.0011)	0.8094(0.0006)	0.6101(0.0026)
	SCAD(41)	0.7147(0.0011)	0.8101(0.0007)	0.6127(0.0026)
	BAR(7)	0.7016(0.0007)	0.8030(0.0005)	0.5925(0.0022)
**EM**	LASSO(20)	0.7140(0.0010)	0.8097(0.0007)	0.6117(0.0028)
	SCAD(30)	0.7148(0.0009)	0.8102(0.0008)	0.6131(0.0024)
	BAR(7)	0.7069(0.0007)	0.8057(0.0006)	0.6010(0.0020)
**KNN**	LASSO(20)	0.7152(0.0008)	0.8104(0.0006)	0.6134(0.0027)
	SCAD(38)	0.7162(0.0011)	0.8106(0.0006)	0.6159(0.0027)
	BAR(7)	0.7070(0.0008)	0.8058(0.0007)	0.6012(0.0024)
**DAE**	LASSO(27)	0.7056(0.0008)	0.7999(0.0009)	0.6160(0.0021)
	SCAD(62)	0.7160(0.0012)	0.8027(0.0009)	0.6412(0.0030)
	BAR(7)	0.6944(0.0008)	0.7944(0.0011)	0.5962(0.0022)
**GAIN**	LASSO(10)	**0.7939(0.0006)**	0.8508(0.0004)	0.7554(0.0014)
	SCAD(52)	**0.8009(0.0008)**	0.8557(0.0006)	0.7643(0.0016)
	BAR(9)	**0.7960(0.0006)**	0.8521(0.0005)	0.7589(0.0015)

**Table 11:** Classification results of datasets after feature selection under SVC

Imputation methods	FSM(No. of selected features)	Accuracy	F1	AUC
**Mean**	LASSO(20)	0.7884(<e-33)	0.8570(1.1093e-31)	0.8338(< e-33)
	SCAD(41)	0.7227(1.1093e-31)	0.8211(1.2325e-32)	0.7798(4.9303e-32)
	BAR(7)	0.6963(4.9303e-32)	0.8209(1.2325e-32)	0.7321(<e-33)
**EM**	LASSO(20)	0.6963(4.9303e-32)	0.8209(1.2325e-32)	0.7408(4.9303e-32)
	SCAD(30)	0.6964( 4.9304e-32)	0.8210(1.2326e-32)	0.7481(4.9304e-32)
	BAR(7)	0.6963(4.9303e-32)	0.8209(1.2325e-32)	0.7311(1.2326e-32)
**KNN**	LASSO(20)	0.7574(1.2325e-32)	0.8369(4.9303e-32)	0.7959(4.9303e-32)
	SCAD(38)	0.7648(4.9304e-32)	0.8408(1.2325e-32)	0.8082(1.1093e-31)
	BAR(7)	0.6963(4.9303e-32)	0.8209(1.2325e-32)	0.7311(1.2326e-32)
**DAE**	LASSO(27)	0.7223(1.1093e-31)	0.8157(<e-33)	0.7845(<e-33)
	SCAD(62)	0.7089( 1.2326e-32)	0.8139(<e-33)	0.7648(1.2325e-32)
	BAR(7)	0.6963(4.9303e-32)	0.8209(1.2325e-32)	0.6851(1.2325e-32)
**GAIN**	LASSO(10)	**0.7971(1.2326e-32)**	0.8524(<e-33)	0.8650(4.9304e-32)
	SCAD(52)	**0.7940((4.9303e-32)**	0.8506(<e-33)	0.8666(<e-33)
	BAR(9)	**0.7980(1.2325e-32)**	0.8528(1.1093e-31)	0.8664(4.9303e-32)

**Table 12:** Classification results of datasets after feature selection under XGBoost classifier

Imputation methods	FSM(No. of selected features)	Accuracy	F1	AUC
**Mean**	LASSO(20)	0.7906(0.0077)	0.8466(0.0060)	0.8586(0.0075)
	SCAD(41)	0.7891(0.0070)	0.8453(0.0058)	0.8581(0.0067)
	BAR(7)	0.7273(0.0077)	0.8114(0.0060)	0.7626(0.0078)
**EM**	LASSO(20)	0.7565(0.0080)	0.8299(0.0064)	0.7991(0.0071)
	SCAD(30)	0.7722(0.0083)	0.8391(0.0066)	0.8275(0.0076)
	BAR(7)	0.7282(0.0071)	0.8125(0.0054)	0.7616(0.0077)
**KNN**	LASSO(20)	0.7714(0.0073)	0.8375(0.0061)	0.8282(0.0079)
	SCAD(38)	0.7879(0.0083)	0.8455(0.0068)	0.8534(0.0074)
	BAR(7)	0.7282(0.0071)	0.8124(0.0054)	0.7616(0.0077)
**DAE**	LASSO(27)	0.6984(0.0086)	0.7859(0.0074)	0.7529(0.0082)
	SCAD(62)	0.7006(0.0089)	0.7867(0.0073)	0.7563(0.0082)
	BAR(7)	0.6866(0.0076)	0.7791(0.0061)	0.7400(0.0090)
**GAIN**	LASSO(10)	**0.7847(0.0066)**	0.8423(0.0056)	0.8522(0.0065)
	SCAD(52)	**0.7895(0.0079)**	0.8455(0.0067)	0.8574(0.0072)
	BAR(9)	**0.7906(0.0077)**	0.8460(0.0062)	0.8572(0.0073)

**Table 13:** Classification results of datasets after feature selection under RF classifier

Imputation methods	FSM(No. of selected features)	Accuracy	F1	AUC
**Mean**	LASSO(20)	0.7995(0.0105)	0.8541(0.0085)	0.7659(0.0119)
	SCAD(41)	0.7945(0.0109)	0.8499(0.0091)	0.7617(0.0122)
	BAR(7)	0.7303(0.0136)	0.8163(0.0102)	0.6451(0.0172)
**EM**	LASSO(20)	0.7558(0.0114)	0.8308(0.0087)	0.6853(0.0133)
	SCAD(30)	0.7645(0.0124)	0.8359(0.0098)	0.6988(0.0137)
	BAR(7)	0.7289(0.0123)	0.8134(0.0098)	0.6500(0.0138)
**KNN**	LASSO(20)	0.7677(0.0111)	0.8375(0.0088)	0.7045(0.0129)
	SCAD(38)	0.7907(0.0118)	0.8485(0.0095)	0.7523(0.0135)
	BAR(7)	0.7306(0.0106)	0.8150(0.0086)	0.6506(0.0124)
**DAE**	LASSO(27)	0.7018(0.0114)	0.7896(0.0095)	0.6339(0.0125)
	SCAD(62)	0.7009(0.0112)	0.7875(0.0091)	0.6372(0.0134)
	BAR(7)	0.6895(0.0135)	0.7810(0.0108)	0.6193(0.0145)
**GAIN**	LASSO(10)	**0.8011(0.0076)**	0.8501(0.0092)	0.7680(0.0127)
	SCAD(52)	**0.8025(0.0099)**	0.8559(0.0081)	0.7692(0.0118)
	BAR(9)	**0.7986(0.0061)**	0.8508(0.0051)	0.7705(0.0078)

**Table 14:** Classification results of datasets after feature selection under LASSO-LR classifier

Imputation methods	FSM(NO.of selected features)	Accuracy	F1	AUC
**Mean**	Lasso(20)	0.7115(0.0075)	0.8086(0.0058)	0.6071(0.0068)
	SCAD(41)	0.7116(0.0078)	0.8086(0.0059)	0.6072(0.0075)
	BAR(7)	0.6997(0.0073)	0.8021(0.0056)	0.5895(0.0067)
**EM**	Lasso(20)	0.7117(0.0074)	0.8086(0.0058)	0.6080(0.0068)
	SCAD(30)	0.7121(0.0075)	0.8086(0.0058)	0.6093(0.0069)
	BAR(7)	0.7058(0.0075)	0.8052(0.0058)	0.5991(0.0069)
**KNN**	Lasso(20)	0.7133(0.0073)	0.8094(0.0056)	0.6105(0.0073)
	SCAD(38)	0.7135(0.0073)	0.8094(0.0057)	0.6114(0.0070)
	BAR(7)	0.7058(0.0075)	0.8053(0.0058)	0.5991(0.0069)
**DAE**	Lasso(27)	0.7022(0.0081)	0.7977(0.0064)	0.6113(0.0084)
	SCAD(62)	0.7080(0.0077)	0.7987(0.0060)	0.6271(0.0082)
	BAR(7)	0.6932(0.0083)	0.7943(0.0068)	0.5926(0.0073)
**GAIN**	Lasso(10)	**0.7921(0.0065)**	0.8495(0.0053)	0.7536(0.0074)
	SCAD(52)	**0.7952(0.0076)**	0.8517(0.0061)	0.7573(0.0084)
	BAR(9)	**0.7943(0.0070)**	0.8508(0.0056)	0.7574(0.0079)

**Table 15:** Classification results of datasets after feature selection under SCAD-LR classifier

Imputation methods	FSM(No. of selected features)	Accuracy	F1	AUC
**Mean**	Lasso(20)	0.7111(0.0077)	0.8095(0.0058)	0.6025(0.0069)
	SCAD(41)	0.7119(0.0081)	0.8098(0.0061)	0.6041(0.0072)
	BAR(7)	0.6992(0.0085)	0.8033(0.0062)	0.5837(0.0073)
**EM**	Lasso(20)	0.7124(0.0080)	0.8103(0.0060)	0.6043(0.0071)
	SCAD(30)	0.7124(0.0078)	0.8102(0.0059)	0.6049(0.0071)
	BAR(7)	0.7063(0.0079)	0.8069(0.0060)	0.5950(0.0067)
**KNN**	Lasso(20)	0.7134(0.0080)	0.8108(0.0060)	0.6069(0.0072)
	SCAD(38)	0.7141(0.0078)	0.8109(0.0059)	0.6083(0.0071)
	BAR(7)	0.7063(0.0079)	0.8069(0.0060)	0.5950(0.0067)
**DAE**	Lasso(27)	0.7009(0.0089)	0.7997(0.0071)	0.6007(0.0078)
	SCAD(62)	0.6945(0.0058)	0.7985(0.0047)	0.5840(0.0060)
	BAR(7)	0.6945(0.0058)	0.7985(0.0047)	0.5840(0.0060)
**GAIN**	Lasso(10)	**0.7899(0.0067)**	0.8500(0.0053)	0.7433(0.0075)
	SCAD(52)	**0.7918(0.0074)**	0.8501(0.0059)	0.7505(0.0081)
	BAR(9)	**0.7908(0.0069)**	0.8514(0.0055)	0.7411(0.0078)

**Table 16:** Classification results of datasets after feature selection under Elastic Net-LR classifier

Imputation methods	FSM(No. of selected features)	Accuracy	F1	AUC
**Mean**	Lasso(20)	0.7143(0.0075)	0.8107(0.0063)	0.6092(0.0064)
	SCAD(41)	0.7115(0.0112)	0.8090(0.8091)	0.6056(0.0098)
	BAR(7)	0.7019(0.0100)	0.8036(0.0077)	0.5916(0.0080)
**EM**	Lasso(20)	0.7148(0.0079)	0.8109(0.0064)	0.6106(0.0063)
	SCAD(30)	0.7135(0.0092)	0.8098(0.0072)	0.6097(0.0080)
	BAR(7)	0.7068(0.0082)	0.8061(0.0063)	0.5996(0.0070)
**KNN**	Lasso(20)	0.7139(0.0078)	0.8099(0.0060)	0.6108(0.0073)
	SCAD(38)	0.7142(0.0076)	0.8101(0.0059)	0.6115(0.0070)
	BAR(7)	0.7068(0.0082)	0.8061(0.0063)	0.5996(0.0070)
**DAE**	Lasso(27)	0.7024(0.0089)	0.7978(0.0066)	0.6115(0.0099)
	SCAD(62)	0.6939(0.0064)	0.7953(0.0054)	0.5921(0.0061)
	BAR(7)	0.6939(0.0064)	0.7953(0.0054)	0.5921(0.0061)
**GAIN**	Lasso(10)	**0.7923(0.0063)**	0.8497(0.0052)	0.7538(0.0071)
	SCAD(52)	**0.7952(0.0069)**	0.8516(0.0058)	0.7580(0.0072)
	BAR(9)	**0.7944(0.0070)**	0.8508(0.0056)	0.7575(0.0079)

**Table 17:** Logistic regression analysis for risk factors of vaginal prolapse

Factors	$\beta ^{a}$	Wald	OR(95% CI)$^{b}$	*P* Value
Intercept	0.09	0.07	1.09(0.58,2.04)	0.79
During pregnancy incontinence	0.34	20.8	1.40(1.21,1.62)	5.10e-06
Stress incontinence	1.24	39.45	3.46(2.38,5.18)	3.36e-10
Vaginal laxity	0.28	36.32	1.32(1.21,1.45)	1.68e-09
Cevix	0.23	62.77	1.25(1.19,1.33)	2.33e-15
Genital hiatus	0.37	111.03	1.45(1.35,1.55)	<2e-16
Perineal body	0.17	23.14	1.18(1.11,1.27)	1.50e-06
Posteri	1.90	621.55	6.72(5.79,7.81)	<2e-16
Fast muscle recovery	0.27	939.62	1.30(1.28,1.33)	<2e-16
Delivery mode	0.35	328.33	1.42(1.37,1.47)	<2e-16

^a^ $$\beta$$ = Coefficient

^b^ OR(95% CI) = Odds Ratio with 95% Confidence Interval

**Fig. 3:**
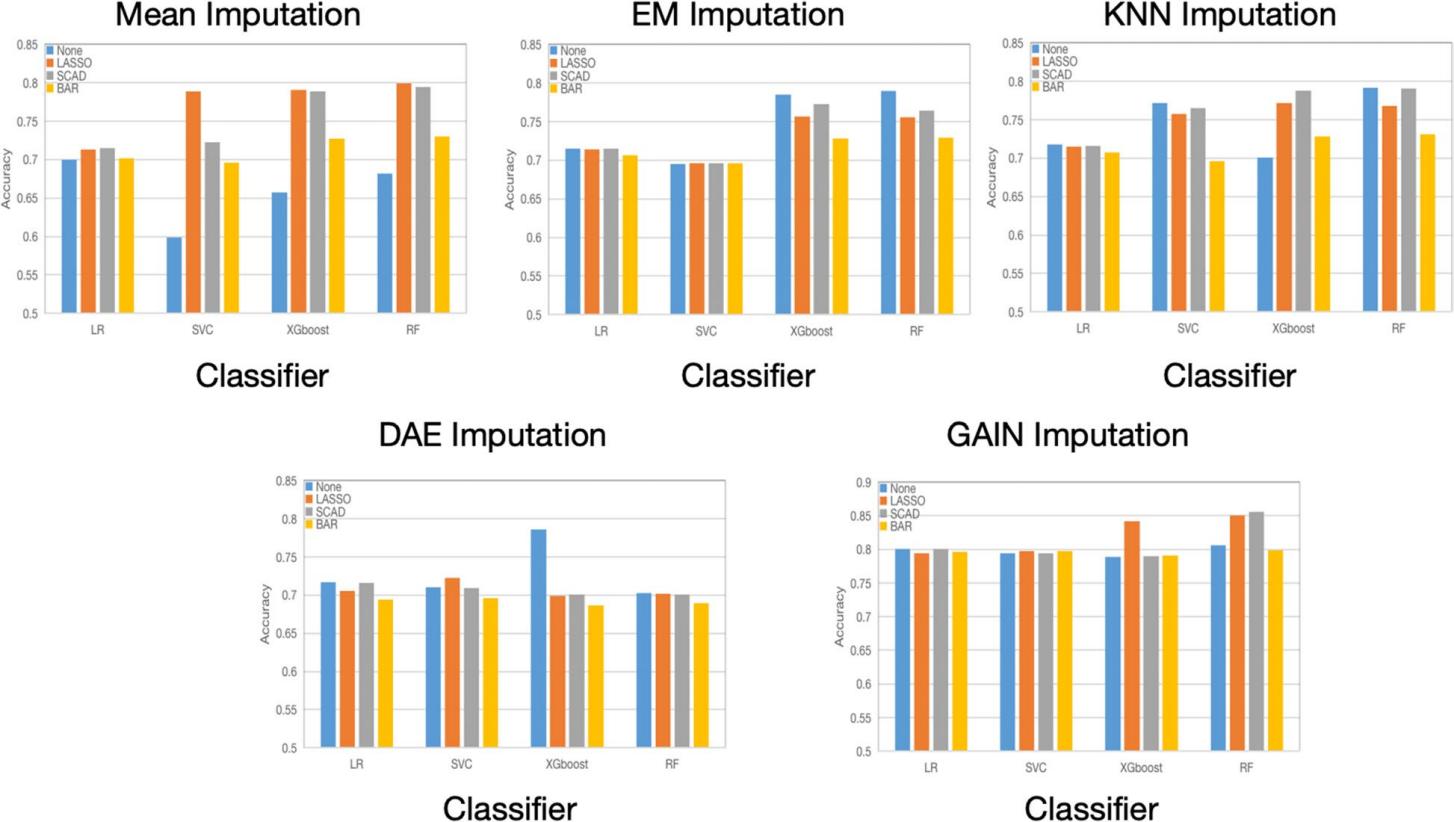
Comparison of classification accuracy before and after feature selection under LR, SVC and RF

As in the previous subsection, we evaluated the performance of the feature selection methods using classifiers mentioned in “Evaluation criteria”. For each classifier, we repeated the experiment 100 times and show the averaged results. Our focus is on examining the changes in prediction ability before and after variable selection, following each imputation. Therefore, LD is not considered in the following comparison. Tables 10, 11, 12, 13, 14, 15 and 16 demonstrates the performance of the three feature selection methods on imputed datasets, which includes the number of selected variables and the classification accuracy of the datasets after variable selection, $F_1$ score and AUC. Regarding the number of variables selected, BAR outperforms LASSO and SCAD by selecting the fewest variables. However, except for the GAIN-imputed dataset, the classification accuracy for LR and RF based other imputed datasets decreases using variables selected by BAR. In contrast, the classification accuracy remains relatively stable after using LASSO and SCAD. While variable selection improves the accuracy of SVC, the overall classification accuracy of SVC remains lower than that of RF. Figure 3 shows the classification accuracy of LR, SVC and RF before and after feature selection. Table 4 and Fig. 3 indicate that using RF as the classifier will result in higher classification accuracy. However, in terms of model performance, GAIN is more stable and performs better. Overall, for the GAIN-imputed dataset, variable selection using the BAR method can largely improve interpretability while maintaining a high classification accuracy. Table 17 gives result of the logistic regression using the 9 selected features on the GAIN-imputed data, all 9 variables are extremely significant with small *P* values.

## Conclusions and discussions

This study aims to provide optimal solutions for the three most common problems in computer-aided medical diagnosis: data loss, accurate prediction, and risk factor analysis. For missing data imputation, two statistical methods (mean imputation and EM imputation) and three machine learning methods (KNN, DAE and GAIN) are considered. The evaluation results show that the GAIN method has the best imputation effect, with a classification accuracy of $81.32\%$. To further enhance the interpretability of our findings, we implement and compare three variable selection methods (LASSO, SCAD, and BAR) on the each imputed dataset. Our results show that BAR feature selection on the GAIN-imputed dataset can improve interpretability with only 9 out of 67 selected factors while maintaining high classification accuracy using the RF classifier.

It is verified that all these 9 selected features are strongly associated with vaginal prolapse. For example, the mode of delivery is a major risk factor to primary POP [48] and prolapse of the anterior vaginal wall is the most common form of POP [1], therefore, it is an important predictor of anterior vaginal prolapse. Additionally, variables such as posteri (Bp), posterior fornix (D), and cervix (C) are consistent with the points for measurement in the Pelvic Organ Prolapse Quantification System (POP-Q) .The Pelvic Organ Prolapse Quantification System (POP-Q) was introduced in 1996 as a standard system for the description of female POP and pelvic floor dysfunction. As the most commonly used pelvic support staging system, POP-Q is approved by the International Continence Society, the American Urogynecologic Society and the Society of Gynecologic Surgeons [49]. Moreover, variables like genital hiatus (gh) and perineal body (pb) are consistent with the landmarks in POP-Q [50]. What’s more, the main etiology of stress incontinence is associated with loss of pelvic support, therefore stress incontinence may predict the occurrence of POP [51]. Overall, the selected features provide valuable insights into the important predictors of vaginal prolapse and can aid in the development of better diagnostic and treatment strategies.

In this dataset, occupations of the patients are only divided into mental and manual workers, so there may be cognitive differences. And the sample size of the dataset is not large enough and needs to be further expanded. Furthermore, some patients are not only with vaginal prolapse, but also may be combined with other pelvic floor diseases such as uterine prolapse or urinary incontinence, resulting in crossing between different diseases. In the future, we will concentrate on how to generalize this framework to wider range of datasets.

## Data Availability

The datasets used and/or analysed during the current study are available from the corresponding author on reasonable request. The code is available at https://github.com/Mingxuan-F/Diagnosis-of-Vaginal-Prolapse.git
